# Establishment, Characterization and Downstream Application of Primary Ovarian Cancer Cells Derived from Solid Tumors

**DOI:** 10.1371/journal.pone.0050519

**Published:** 2012-11-30

**Authors:** Thanasak Sueblinvong, Rahel Ghebre, Yoshie Iizuka, Stefan E. Pambuccian, Rachel Isaksson Vogel, Amy P. N. Skubitz, Martina Bazzaro

**Affiliations:** 1 Departments of Obstetrics, Gynecology and Women’s Heath, University of Minnesota, Minneapolis, Minnesota, United States of America; 2 Department of Laboratory Medicine and Pathology, University of Minnesota, Minneapolis, Minnesota, United States of America; 3 Masonic Cancer Center, University of Minnesota, Minneapolis, Minnesota, United States of America; University of Edinburgh, United Kingdom

## Abstract

Ovarian cancer is the deadliest of the gynecological diseases and the fifth cause of cancer death among American women. This is mainly due to the lack of prognostic tools capable of detecting early stages of ovarian cancer and to the high rate of resistance to the current chemotherapeutic regimens. In this scenario the overall 5-year survival rate for ovarian cancer patients diagnosed at late stage is less than 25%. Abnormalities associated with the malignant phenotype and the mechanisms of tumor progression are not clearly understood. *In vitro* studies are necessary, yet have been hampered due to the limitations accompanied with the use of ovarian cancer cell lines and the heterogeneity of the ovarian cancer cell population derived from ascites fluids. In this study we present a simple, rapid and reproducible method for the isolation and characterization of ovarian cancer cells from solid tumor tissue and show that enzymatic digestion for 30 minutes with dispase II results in the most effective recovery of viable epithelial ovarian cancer (EOC) cells. The resulting cancer (EOC) cell preparations demonstrate a significant yield, high levels of viability and are fibroblast-free. They grow for up to six passages and retain the capacity of forming spheroids-like structures in agarose. In addition, they can be genetically manipulated and used for drug screening, thus rendering them highly suitable for downstream applications. Notably, isolation of ovarian cancer cells from solid specimens using this method has the advantage of allowing for isolation of cancer cells from early stages of ovarian cancer as well as obtaining cells from defined either primary and/or metastatic ovarian cancer sites. Thus, these cells are highly suitable for investigations aimed at understanding ovarian cancer.

## Introduction

Morphologic and molecular genetic studies have established that ovarian cancer is a heterogeneous disease that encompasses a number of different histotypes including high-grade serous carcinoma [1]. The current regimen of chemotherapy for ovarian cancer consists of taxane and platinum-based therapy. While >80% of patients with early stage disease show a 5 year survival, the treatment has limited efficacy against advanced-stage epithelial ovarian cancer (EOC). The lack of effective diagnostic and prognostic tools for ovarian cancer renders this particular cancer extremely difficult to manage and most patients develop chemotherapy resistant tumors and relapse [2], [3], [4], [5], [6]. Much of our current knowledge of human ovarian cancer has been uncovered through the use of established immortalized ovarian surface epithelial cells (IOSEs), ovarian cancer cell lines and primary ovarian cancer cells derived from ascitic fluids [7], [8]. The use of IOSEs and ovarian cancer cell lines has obvious advantages including their high proliferative capacity and extended lifespan. Unfortunately, both the genetic and phenotypic changes associated with extended *in vitro* passages and the heterogeneity resulting from primary ovarian cancer cells from ascitic fluids makes them a less than ideal model for ovarian cancer studies. Thus, the ability to isolate and culture fresh EOC cells from solid specimens ovarian cancer provides a unique model for studies of new therapeutic approaches for ovarian cancer treatment.

**Table 1:** Donors characteristics.

Donor	Age	Type of EpithelialOvarian Cancer	Site	Grade	Stage
1	56	Borderline serous	Ovary	–	IB
2	66	Borderline serous	Ovary	–	IB
3	47	Serous adenocarcinoma	Ovary	3	IIIC
4	57	Undifferentiated high grade	Omentum	3	IV
5	61	Serous adenocarcinoma	Omentum	3	IV
6	63	Serous adenocarcinoma	Omentum	3	IIIC
7	63	Serous adenocarcinoma	Omentum	3	IIIC
8	68	Serous adenocarcinoma	Ovary	3	IIIC

Several methods have been described for the primary culture of either human ovarian surface epithelial (OSE) cells from normal ovaries or EOC cells isolated from the ascites fluid of cancer patients [9], [10], [11], [12], [13], [14], [15]. However, the growth of malignant cells from solid tumors has been problematic because of stromal cell or fibroblast overgrowth, little to no growth of malignant cells, or premature loss of proliferative capacity in culture. While there are several options to create single-cell suspensions from solid tumors through mechanical means or enzymatic dissociation, no one has addressed which technique yields the most reliable outcome [16], [17], [18]. In this study, we describe for the first time a systematic comparison between mechanical disruption and enzymatic digestion with either collagenase A, hyaluronidase or dispase II for various amount of time from 30 to 120 minutes for the isolation of EOC cells from solid ovarian cancer tumors. We report that enzymatic digestion for 30 minutes with dispase II results in the most effective recovery of viable EOC cells and that prolonged exposure to enzymatic digestion is accompanied with significant decrease in the recovery of viable cells.

The EOC cell cultures are fibroblast-free, as evaluated by EpCAM staining with the MOC-31 and Ber-EP4 mAbs, and grew exponentially for up to six passages before they senesced and died. Importantly, the EOC cultures retained some of the phenotypical characteristics of the tumors from which they originated, including the capacity to form spheroid-like structures on agar substrates. Lastly, the EOC cultures were suitable for downstream applications as they were highly susceptible to genetic manipulation by means of lentiviral infection and useful in drug screening tests.

**Figure 1:**
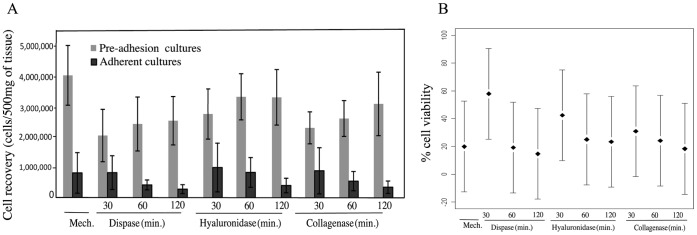
EOC cell isolation by different methods of cell dissociation. **A.** Mean ± standard deviation of total number of viable cells per 500 mg of tissue derived from each condition immediately after treatment (i.e. pre-adhesion cultures) and then again after 7–10 days of adhesion in tissue culture (i.e. adherent cultures). B. Percentage of cell viability for each condition expressed as percentage of pre-adhesion cultures to adherent cultures. Least-square means (adjusted for replicates within patient) of eight independent experiments and the 95% confidence intervals are presented.

## Materials and Methods

### Materials

The following materials were purchased from Invitrogen (Carlsbad, CA) Cell culture media: DMEM, fetal bovine serum (FBS), 100X penicillin-streptomycin, 0.05% w/v trypsin-EDTA. Enzymes: dispase II and collagenase A (Roche, Indianapolis, IN), hyalorunidase (EMD Chemicals, Gibbston, NJ).

**Figure 2:**
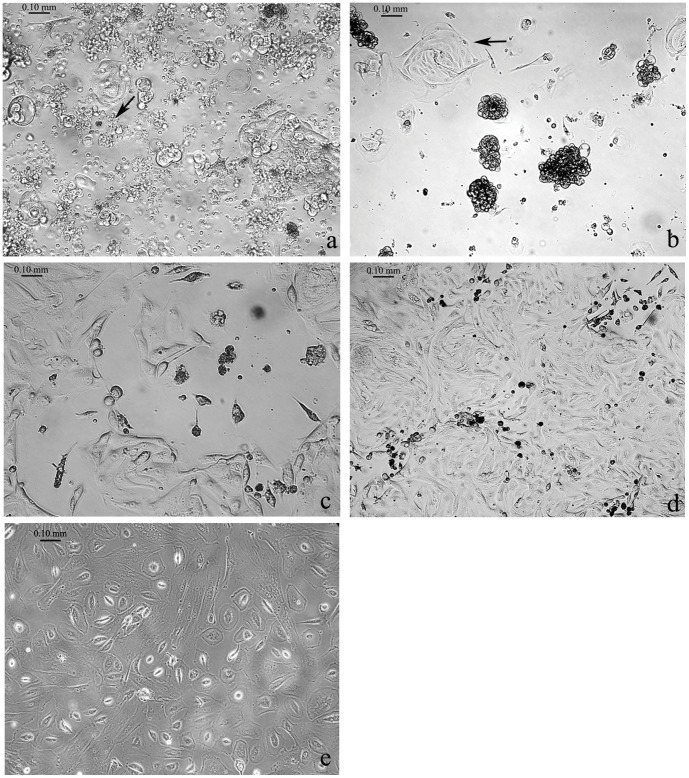
Morphologic characteristics of EOC cells. (a) Representative examples of primary EOC cells isolated from cancer specimen after two days in culture (arrow indicates erythrocytes); (b) Swirl-like clusters of EOC cells after 3–4 days in culture (arrow indicates the growing clusters); (c) A colony of EOC cells spreading on the tissue culture plastic after 7–8 days in culture; (d) A confluent monolayer of EOC cells depicting typical epithelial cobblestone morphology after 13–14 days in culture; and (e) A confluent monolayer of fibroblasts here used as negative control.

#### Antibodies

The two monoclonal antibodies that recognize EpCAM, Ber-EP4 and MOC-31, were purchased from Dako (Carpinteria, CA) and used at the concentration recommended by the manufacturer for immunohistochemical analysis. The choice of using these two particular anti-EpCAM mAbs was made because they have shown greater specificity for epithelial cells as compared to other epithelial marker [19], [20], [21], [22] The ICC of primary EOCs cultures was performed by the certified technicians in the Immunocytochemistry and Histopathology Laboratory of Fairview University Medical Center at the University of Minnesota where these markers are routinely used for staining of surgical specimens to confirm their epithelial nature, including ovarian cancer clinical specimens. The PARP, β-actin and peroxidase-linked anti-mouse and anti-rabbit Immunoglobulin G antibodies were purchased from BD Pharmingen (San Diego, California) and Sigma (St. Louis, MO) respectively and utilized at the concentration recommended by the manufacturer for immunoblot analysis.

#### Cell lines

The human ovarian cancer cell lines ES-2 and NIH:OVCAR5 were was purchased from the American Type Culture Collection (Manassas, VA).

**Figure 3:**
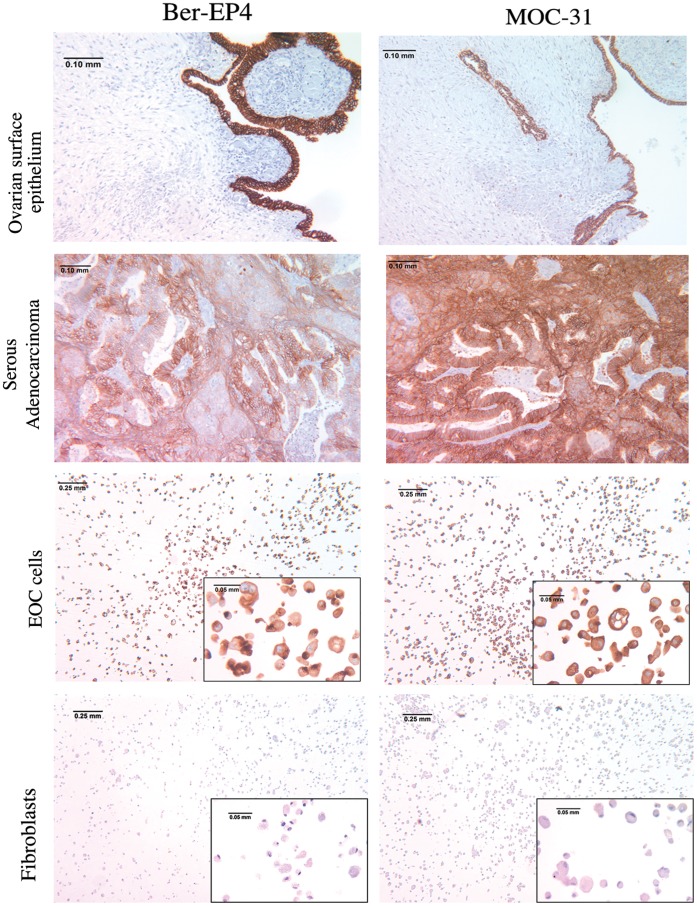
Immunostaining of clinical specimens and freshly isolated EOC cells for EpCAM expression. A FFPE block of normal ovary, depicting positive staining for EpCAM with both the monoclonal antibodies Ber-EP4 and MOC-31 of the normal ovarian surface epithelial cells. A FFPE block of serous ovarian adenocarcinoma depicting positive staining for EpCAM with both the monoclonal antibodies Ber-EP4 and MOC-31 of the EOC cells within the tumor. Immunocytochemical staining of paraffin-embedded EOC cultures with the monoclonal antibodies Ber-EP4 and MOC-31 depicting positive staining for EpCAM. Immunocytochemical staining of paraffin-embedded fibroblast cultures with the monoclonal antibodies Ber-EP4 and MOC-31, here used as negative controls.

### Reagent Setup

#### Complete DMEM medium

DMEM was supplemented with 10% FBS, and 100 units penicillin-streptomycin. The medium was stored at 4°C and warmed to 37°C prior to use.

#### Enzymes

Dispase II (2.4 U/ml), hyaluronidase (0.0369 U/ml) and collagenase A (0.15 U/ml) were diluted in PBS, aliquoted and stored at −20°C in a non-frost-free freezer.

**Figure 4:**
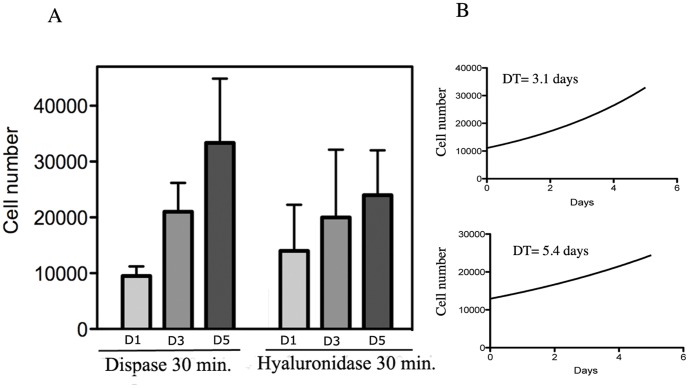
Proliferation rate of EOC cells. A. Cultures of EOC cells (donor 8) derived from 30 minutes of enzymatic digestion with dispase II or hyaluronidase were evaluated for their proliferation over a period of 5 days (D1, D3, D5). Experiments were conducted in triplicates. B. Doubling time for cells treated with dispase II for 30 minutes (*top*) or hyaluronidase 30 minutes (*bottom*) calculated by non linear regression analysis.

#### Tissue collection

Eight tissue samples (∼5 g in size) from EOC tumors were obtained from the University of Minnesota Tissue Procurement Facility (TPF) after Institutional Review Board Committee: Human Subject Board (IRB) approval. Specifically, consent was waived by the IRB of the University of Minnesota.

The patients’ ages ranged between 47 and 68 years old. The average patient age was 60. Tissue samples were collected from areas macroscopically identified as cancer by pathologists. Samples were deidentified and registered following the protocol of the BioNet Tissue Procurement Facility. Selected tissue samples were placed into sterile 50-ml conical tubes containing ice-cold DMEM, which were then delivered to a cell culture laboratory in a bucket of ice within 30 minutes of fresh sample collection.

Adjacent areas of all tumors underwent subsequent routine tissue processing (formalin fixation and paraffin embedding). Histopathologic analysis of sections to assess the sub-type, grade of ovarian cancer and staging based on pathologic and imaging data performed by surgical pathologists at the University of Minnesota Medical Center, Fairview.

**Figure 5:**
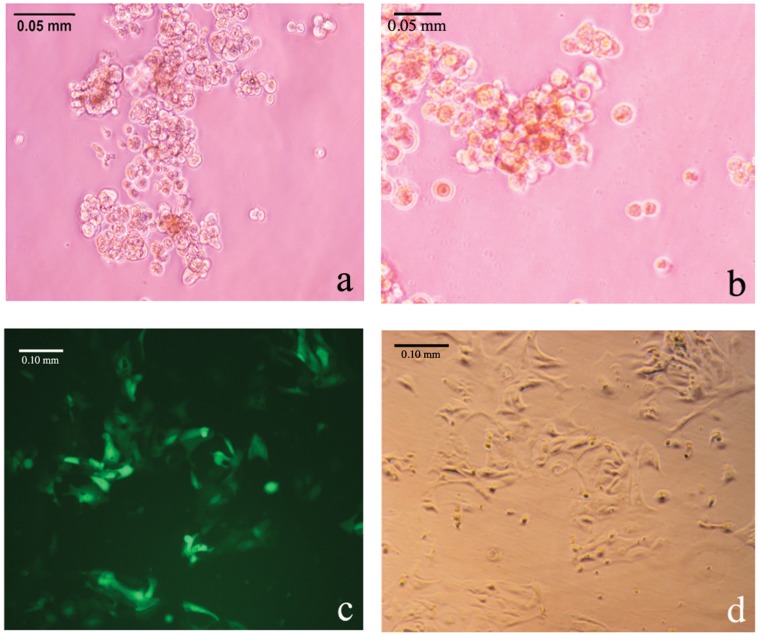
Down-stream application of EOC cells for anchorage independent growth and transduction efficiency. (a) EOC cells from donor 8 forming spheroids-like structures on agarose; (b) NIH:OVCAR5 human ovarian cancer cell line also formed spheroids (positive control); (c) EOC cells from donor 8 transfected with the GFP expressing lentivirus pEF-GFP-SIN; and (d) phase contrast of the same field.

#### Epithelial Ovarian Cancer (EOC) isolation protocol

Tumor specimens (∼5 g) were divided with a scalpel into ten pieces of equal weight (∼500 mg) that were placed in 60 mm Petri dishes containing 5 ml of DMEM and further cut in smaller pieces of approximately 2 mm. For mechanical disruption, the specimen slurry was pressed through a 70 µm mesh with a syringe plunger and the cell filtrate was centrifuged at 1200 rpm for 7 minutes at 4°C. The remaining nine samples were processed via enzymatic digestion by adding either collagenase A, hyaluronidase or dispase II, then incubated for 30 to 120 minutes, at which time the cell slurries were passed through the 70 µm mesh and centrifuged as described above. After resuspension in DMEM containing 10% FBS, samples for each condition were evaluated for cell viability by trypan blue dye exclusion. Medium was changed 24 hours after initial plating and every three days for the following two weeks.

#### Immunocytochemical analysis

Immunocytochemical analysis for EpCAM expression with the monoclonal antibodies MOC-31 and Ber-EP4 was performed on ovarian cancer cells that were in culture for 14 days. Specifically, primary ovarian cancer cells were harvested by trypsinization, washed with PBS and spun. The derived cell pellet was subsequently formalin-fixed and paraffin embedded (FFPE EOC) to generate cell blocks. Sections were subjected to immunocytochemistry using the Ventana XT automated stainer from Ventana Medical System (Tucson, Arizona). Briefly, slides were deparaffinized and following antigen retrieval, incubated with the monoclonal antibodies against EpCAM (MOC-31 and Ber-EP4) for 32 and 16 minutes, respectively, before staining with the iVIEW DAB detection kit from Dako (Carpinteria, CA). Immunocytochemistry was performed in the Histopathology Laboratory of Fairview University Medical Center at the University of Minnesota.

#### Measurement of cell viability

Cell number and viability of the EOC cultures was determined by the trypan blue exclusion assay.

#### Anchorage independent assay

The method used for the anchorage independent assay was based on the liquid overlay technique as previously described [23]. Briefly, to prohibit cell adhesion to a substratum, 24-well tissue culture plates (Becton Dickinson, Franklin Lakes, NJ) were coated with 200 µl of 0.5% SeaKem LE agarose (BioWittaker Molecular Applications, Rockland, ME) in serum-free media, and allowed to solidify for 30 minutes at 20°C. NIH:OVCAR5 or EOC cell suspensions (5,000 cells) were layered onto the top of the solid agarose-coated plates at a volume of 1 ml/well in DMEM supplemented with 10% FBS and then incubated for 48 hours at 37°C.

#### Morphology of cells and spheroids

A Nikon Eclipse TE 2000E inverted microscope was used for the imaging of cellular morphology with phase contrast. Images were captured with Spot 3.5.8 acquisition software (Diagnostic Instruments, Sterling Heights, MI).

#### Infection of EOC with lentivirus

The lentivirus vector pEF-GFP-SIN, the VSVG envelope and the delta-8.9 plasmids were co-transfected into the 293T cells for virus production as previously described [24]. The virus supernatant was spin-infected in the presence of 10 µg/ml of polybrene to 1×10^5^ of the EOC cells at 2500 rpm for 2 hrs at 33°C. GFP expression was monitored 48 hours after the infection.

#### Western blot analysis

Total cellular protein (10–20 µg) from each sample was separated by SDS-PAGE, transferred to PVDF membranes and subjected to Western blot analysis using PARP and β-actin antibodies.

**Figure 6:**
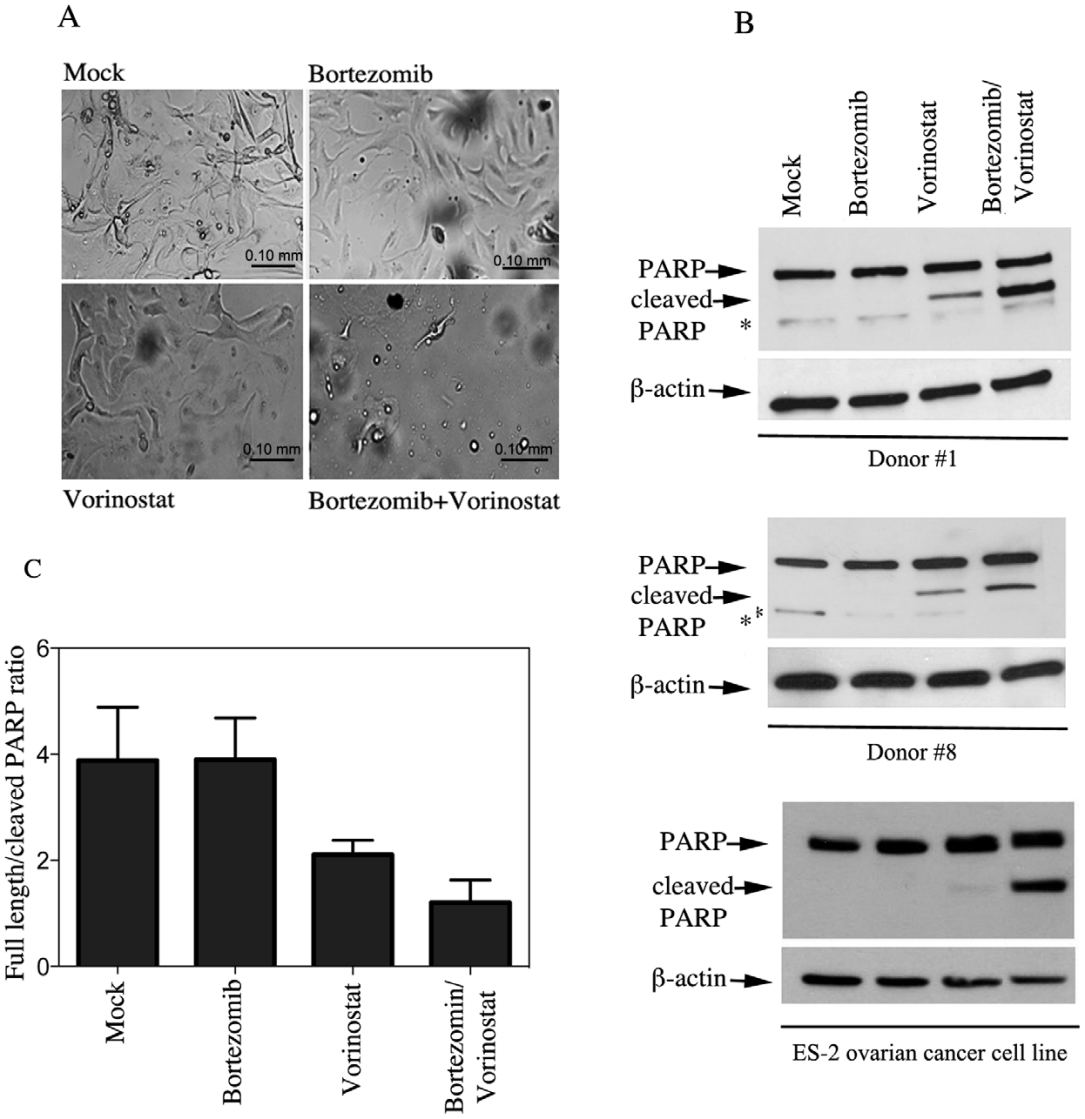
Down-stream application of EOC cells for drug toxicity studies. A. Morphological changes in EOC cells either mock treated or treated with Bortezomib (2 nM) and Vorinostat (6 µM) alone and in combination for 18 hours. B. Representative examples of lysate of EOC cells from donors 1 and 8 (*top and middle panel*) or ES-2 human ovarian cancer cell line (*bottom panel*) either mock treated or treated with Bortezomib (2 nM) and Vorinostat (6 µM) or Bortezomib (6 nM) and Vorinostat (3 µM) respectively, alone and in combination for 18 hours and immunoblotted with an antibody recognizing the full-length and the cleaved forms of PARP. Equal protein loading was verified by using an antibody directed against β-actin. * = a specific band. C. Increase in the cleaved-PARP species in the mock versus treated EOC cells expressed as cleaved full length/cleaved PARP ratio.

### Statistical analysis

All outcomes were analyzed using mixed effects linear regression models with fixed effects for treatment or length of treatment as appropriate and a random effect for tumor source. Despite the non-normality of some of the data, linear regression models are quite robust and allow for inclusion of random effects. Least-squares means and standard errors were reported and p-values were adjusted for multiple comparisons using a Bonferroni correction. All significance levels were set at 0.05. Calculation of the cells’ doubling time (DT) was obtained by nonlinear regression analysis using Prism (V.5 Graphpad, San Diego, CA).

## Results and Discussion

### Comparison of Dissociation Techniques

While mechanical disruption and enzymatic digestion represent two obvious choices for the isolation of EOC cells from solid tumor specimens, a direct comparison has not been made to determine which procedure results in a greater cell yield and viability. To address this question, eight solid ovarian cancer clinical specimens including five high grade serous adenocarcinomas, two serous borderline and one undifferentiated high grade carcinomas (Table 1) were collected at the time of surgery and subjected solely to mechanical disruption or to enzymatic digestion followed by mechanical disruption. Specifically, each specimen was cut into samples of equal weight (500 mg) and then subjected solely to mechanical disruption through the use of a 70 um cell sieve or were enzymatically digested for 30, 60 or 120 minutes with dispase II, hyaluronidase or collagenase A and then mechanically disrupted by passage through a 70 µm sieve. For each condition, cell number and viability were assessed by trypan blue dye exclusion at two time points: immediately after specimen processing (pre-adhesion cultures) and after a period of seven to ten days (adherent cultures).

The initial number of cells recovered (i.e. pre-adhesion cultures) was highest when tumors were solely mechanically disrupted without any enzymatic treatments. Not surprisingly, the total number of cells initially recovered following enzymatic treatment increased with the length of time that the tissue was exposed to the various enzymatic treatments. The highest percentage of cell recovery, based on the starting number of cells, occurred when cells were exposed for 30 minutes to dispase II (Figure 1A). While not statistically significant after adjustment for multiple comparisons, the mean percent cell recovery when cells were exposed to dispase II for 30 minutes (58.0%) is higher than the others, most notably mechanical (19.9%; Figure 1B). Interestingly, a week after plating (adherent cultures) those tissues that had been exposed for the longest periods of time to the enzymatic treatment exhibited a statistically significant decrease in the recovery of viable cells (p = 0.02).

Taken together, this suggests that mechanical disruption and prolonged enzymatic treatment were particularly harsh on EOC cells and that a 30 minute exposure to dispase II represents the best choice for obtaining viable EOC cultures.

### Growth Characteristics of EOC Cultures and Characterization of their Epithelial Nature by EpCAM Immunostaining

Next, we documented the morphological changes and growth properties of freshly isolated EOC cells derived from solid tumors. Two days after plating in DMEM supplemented with 10% FBS, EOC cells began to adhere to the plate as indicated by the presence of small adherent clusters (Figure 2a). At day 2, erythrocytes (arrow) still appeared to be viable in the culture. By day 4 (Figure 2b), the adherent colonies of EOC cells formed swirl-like shapes (arrow) and started to spread in the tissue culture plate while the remaining erythrocytes progressively disappeared from the culture. Large multicellular aggregates were also observed at this stage; these later disaggregated into cellular monolayers. By day 7, the initial EOC cell clusters started to proliferate (Figure 2c). By day 14, the EOC cells became a confluent monolayer and displayed the typical epithelial cobblestone morphology (Figure 2d), which appeared to be dissimilar from the one of a 14-day fibroblast culture here used as a negative control (Figure 2e).

To confirm the epithelial nature of the 14-day EOC cultures, we next performed immunocytochemistry (ICC) for the epithelial marker EpCAM using two different monoclonal antibodies, Ber-EP4 and MOC-31. Importantly, unlike the routinely used makers, which cannot distinguish between epithelial cells, mesothelial cells or fibroblasts, the Ber-EP4 and MOC-31 antibodies exclusively stain for the EpCAM marker in epithelial cells [19], [20], [21], [22]. Staining with these two antibodies allowed us to assess the purity of the isolated EOC cells. As shown in Figure 3, EOC cells displayed the characteristic cytoplasmic staining EpCAM with both, Ber-EP4 and MOC-31 monoclonal antibodies. The EOC cells that we isolated were comprised of a homogeneous population of epithelial cells. No difference in terms of purity was observed between mechanical disruption or enzymatic digestion techniques (not shown). As positive controls, normal ovarian surface epithelial (NOSE) cells and serous adenocarcinoma also stained for the epithelial marker EpCAM with both, Ber-EP4 and MOC-31 mAbs Figure 3). A two week-long fibroblastic culture was also subjected to ICC with for the epithelial marker EpCAM with Ber-EP4 and MOC-31 mAbs as negative control (Figure 3).

### Growth Characteristics of EOC Cultures and their Down-stream Applications

In order to evaluate the growth properties of the obtained primary EOCs cultures, we measured the proliferation rate of the EOCs cells that were derived from 30 minutes digestion with dispase II and hyaluronidase approximately at day 14 from isolation (after they became a confluent monolayer, displayed typical epithelial cobblestone morphology and positive staining for the EpCAM epithelial marker). Cells were monitored for proliferation rate by trypan blue dye exclusion counting over the course of seven days. As shown in Figure 4A, EOC cells from both dispase II and hyaluronidase treatments grew with a doubling time (DT) of 3.1 and 5.4 days respectively as calculated by non linear regression analysis (4B *top* and *bottom*). Furthermore, cells obtained by these two enzymatic digestion methods could be passaged up to six times over a period of 4 weeks before they senesced and died (not shown).

A unique model for studying ovarian cancer progression as well as testing the sensitivity of ovarian cancer cells to new chemotherapeutic agents *in vitro* is dependent upon their capacity to form spheroids [23], [25]. In the peritoneal cavity of patients with ovarian cancer, spheroids naturally form in the ascites fluid and, when formed *in vitro*, they mimic the cancer’s three dimensional structure in the tumor itself. Thus we tested the primary EOCs cultures for their ability to form spheroid-like structures in an anchorage independent growth assay by culturing them on the nonadhesive agarose substrate. The ovarian cancer cell line NIH:OVCAR5, which is known to form spheroids, was used as positive control [26]. EOC cells and NIH:OVCAR5 cells were equally capable of forming spheroid-like structures over seven days (Figure 5a and 5b, respectively). The spheroids of multi-cellular aggregates were of varying sizes and were comprised of dozens up to nearly one hundred cells per aggregate.

Genetic manipulation of primary ovarian cancer cells is often required for the understanding of the role of oncogenes and tumor suppressor genes during ovarian cancer initiation and progression. Therefore, we tested the primary EOCs cultures for their ability to be genetically manipulated via infection with lentiviral particles expressing pEF-GFP-SIN. The efficiency of infection was >80% when we measured the ratio between GFP-expressing cells (Figure 5c, blue channel) versus total cell number (Figure 5d, bright field). These experiments provide proof that the isolated primary EOCs cultures cells retained some of their phenotypical characteristics/attributes.

Toxicity studies aimed to determine the activity profile of new chemotherapic agents and their combination in cancer cells is of paramount importance for targeted therapies for ovarian cancer. We have previously shown that the combination of proteasome and pan-HDACs inhibitors induce synergistic killing of ovarian cancer cell lines while sparing their immortalized counterpart [27]. Thus, we compared the sensitivity to this particular drug combination in the primary EOCs cultures as well as in the commercially available ovarian cancer cell line ES-2. Specifically, EOCs cells were exposed to sub-optimal concentrations of the proteasome inhibitor Bortezomib (2 nM) and the pan-HDACs inhibitor Vorinostat (6 µM) either alone or in combination and onset of apoptosis was measured by monitoring morphological changes and expression levels of cleaved PARP in primary EOCs cultures and ES-2 ovarian cancer cell line (from donors 1 and 8) after 18 hours of drug exposure.

As shown in Figure 6A, combination treatment resulted in greater apoptosis-associated morphological changes in primary EOCs cultures as compared to single agent exposure. Combination treatment also resulted in increased levels of cleaved PARP as compared to single agent exposure in both primary EOCs cultures (Figure 6B *upper and middle panel*) and ES-2 ovarian cancer cell lines (Figure 6B, *bottom panel*). Quantification of the ratio between cleaved full length and cleaved PARP is primary EOCs cultures is shown in Figure 6C.

### Conclusions

A better understanding of the initiation, development and progression of ovarian cancer is of paramount importance for improving the outcome of ovarian cancer patients. One of the most challenging problems associated with these studies is the lack of reliability in using established ovarian cancer cell lines, which have often undergone hundreds of passages *in vitro*. The cell lines may no longer resemble the cancer from which they were originally derived. In this study we describe the most efficient and rapid method for isolating EOC cells from solid tumors. The EOC cell preparations are devoid of fibroblasts and grow exponentially for up to 6 passages before they senesce and die. Importantly, these freshly isolated EOC cells retain the capacity to form spheroid-like structures, can easily be transfected by means of a lentiviral system, and are a viable tool for drug screening. Thus, these cells are rendered suitable for downstream research applications.

Lastly, the ability to isolate and manipulate ovarian cancer cells derived from solid tumors versus ascites fluid has the potential advantage of isolating EOC cells from early stages of the disease prior to the time when ascites fluid formation occurs. In particular, cell lines derived from ascites fluid are obtained from a relatively late stage of the disease and it is not possible to determine whether the cells were derived from the primary tumor site or from a metastatic site.
